# The ecological consequences of a pandemic

**DOI:** 10.1098/rsbl.2020.0641

**Published:** 2020-11-18

**Authors:** Julia C. Buck, Sara B. Weinstein

**Affiliations:** 1 Department of Biology and Marine Biology, University of North Carolina Wilmington, 601 S. College Road, Wilmington, NC 28409, USA; 2 School of Biological Sciences, University of Utah, 257 South 1400 East, Salt Lake City, UT 84112, USA

**Keywords:** pathogen, non-consumptive effect, trait-mediated indirect effect, consumer-resource dynamics

## Abstract

The COVID-19 pandemic has altered human behaviour in profound ways, prompting some to question whether the associated economic and social impacts might outweigh disease impacts. This fits into a burgeoning ecological paradigm suggesting that for both predator–prey and parasite–host interactions, non-consumptive effects (avoidance) can be orders of magnitude stronger than consumptive effects (sickness and death). Just as avoidance of predators and parasites imposes substantial costs on prey and hosts, altered behaviour to reduce the transmission of COVID-19 has impacted human fitness and wellbeing. But the effects of infectious disease avoidance do not stop there; non-consumptive effects of predators and parasites often trigger cascading indirect effects in natural systems. Similarly, shifts in human behaviour due to COVID-19 have triggered myriad indirect effects on species and the environment, which can be positive, negative or neutral. We urge researchers to recognize that the environmental impacts associated with lockdowns are indirect effects of the virus. In short, the global response to COVID-19 suggests that the non-consumptive effects of a pathogen, and resulting indirect effects, can be profound.

## 1. Introduction

Less than two weeks after the World Health Organization declared COVID-19 to be a pandemic, Donald Trump tweeted ‘WE CANNOT LET THE CURE BE WORSE THAN THE PROBLEM ITSELF’. Setting aside questions of scientific and semantic accuracy for now, this statement seeks to compare the costs of infection versus infectious disease avoidance. Similar comparisons are often used to understand the behaviour of prey when faced with predators. Although predators are the prototypical consumers, parasites (i.e. all infectious agents; [1]) are increasingly recognized to have parallel effects on their hosts [2]. Both types of natural enemies affect victims through death and/or sickness (consumptive effects), and by causing victims to invest resources to avoid being consumed (non-consumptive effects).

Whereas wild animals routinely balance predation/infection risk and costly risk avoidance [3], due to predator extirpation and disease control, humans rarely face similar decisions. However, when a novel, deadly pathogen emerges for which we lack an effective treatment or vaccine, avoiding infection becomes our best option to minimize morbidity and mortality. We are unique among animals in terms of social organization, communication and technology, and therefore, we have far greater potential to avoid (control) infectious diseases at large scales via government mandates and public health policies. However, if pathogens are not effectively controlled at large scales, individuals are forced to balance infection risk and costly risk avoidance, just like other animals.

## 2. Direct effects

Risk avoidance is widely documented in nature. In the presence of predators, prey often alter their behaviour, morphology, physiology or development to reduce their risk of being eaten. Such non-consumptive (or ‘risk') effects can be costly for prey. In fact, predator avoidance can have a larger impact on prey than does predation ([4] but see [5]). It is increasingly recognized that parasites can have similar, potentially costly, non-consumptive effects on their hosts [6,7]. For instance, spiny lobsters avoid sheltering with conspecifics infected by a deadly virus, thereby increasing predation risk [8,9]. However, although numerous examples of parasite avoidance exist (reviewed by Buck *et al*. [7]), evidence that parasites' non-consumptive effects can outweigh their consumptive effects is lacking. Nevertheless, just as the fiercest predators elicit the greatest response from vulnerable prey, so too should highly contagious, deadly diseases produce large responses from vulnerable hosts.

**Figure 1.**
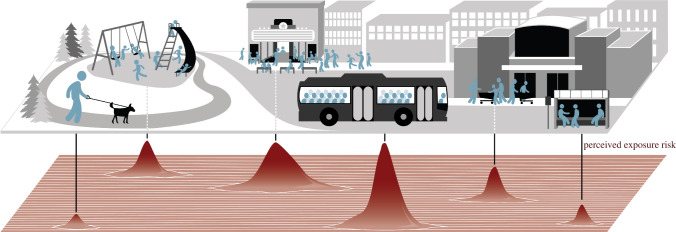
COVID-19 transmission risk is highly heterogeneous and concentrated in areas with more people and less airflow. Perceived infection risk generates a landscape of disgust, akin to the predator-induced landscape of fear. In this landscape, humans alter their behaviour to avoid peaks of risk, generating myriad ecological effects. Artwork: Melissa Smith.

As COVID-19 gained recognition as a highly contagious and deadly pathogen, humans began altering their behaviour to reduce exposure risk (figure 1). These massive behavioural shifts (dubbed the ‘Anthropause'; [10]) were driven by both individual risk perception and government mandates. By late March, one in five humans globally were under lockdown [11], rising to one in three by late April [12]. Although most government-mandated restrictions were eased by July, many people continued to avoid contact with others, especially where the virus was not contained [13]. These measures unquestionably reduced the consumptive effects of the virus by preventing illness [14] and saving lives [15], but pathogen avoidance has proven to be costly for humans.

**Figure 2.**
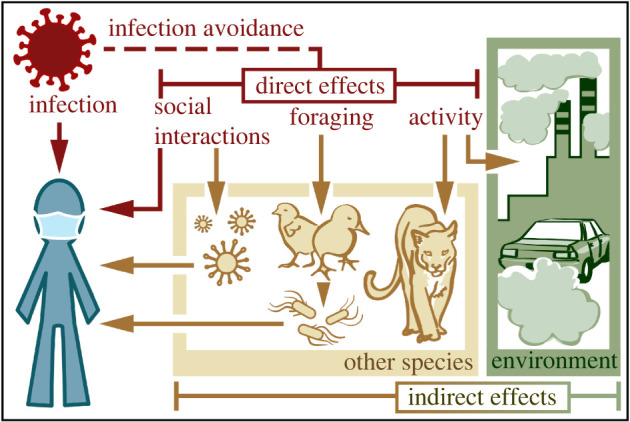
SARS-CoV-2 has consumptive (infection) and non-consumptive (infection avoidance) effects on humans. To avoid infection, humans altered their social interactions, food acquisition and activity patterns (direct effects). These behavioural changes also triggered indirect effects on other species (e.g. fewer mountain lions killed by cars) and the environment (e.g. reduced air pollution), including feedback loops to humans that are positive (e.g. reduced flu transmission from reduced social interaction) and negative (e.g. *Salmonella* outbreaks from increased poultry contact).

The direct effects of COVID-19 on human activity are numerous, including changes in social interactions, movement and food acquisition (figure 2). For example, to reduce exposure risk, humans significantly curtailed both formal and informal social interactions. Schools, workplaces and entertainment venues closed, while parties, nursing home visits and sporting events were cancelled. Infection avoidance reduced human activity so dramatically that it caused the largest seismic noise reduction ever recorded [16]. At peak lockdown, average mobility in the USA declined 55–70% [17] and global surface transport and aviation declined by an estimated 50% and 75%, respectively [18]. Finally, COVID-19 changed how and where people eat, with people consuming more meals at home and selecting options to minimize contact. Changes in purchasing patterns and an abrupt increase in demand for essential items produced shortages and triggered hoarding [19]. Locally sourced foods and home-grown or homemade options (e.g. CSA boxes, gardens, backyard poultry) also gained popularity.

In animal systems, the costs of risk avoidance are often measured in terms of fitness changes (i.e. reproduction, growth rate, condition, mortality) [5,20]. Could the non-consumptive effects of COVID-19 also alter human fitness? Infection avoidance has already resulted in measurable reductions in productivity. As unemployment skyrocketed, reduced income also reduced spending, triggering business closures and job losses [21]. The poorest and most vulnerable among us have disproportionately borne these costs, exacerbating poverty on a global scale [22]. Overall, the lost GDP is expected to exceed 5 trillion globally in 2020 [23]. There is also increasing evidence that COVID-19 avoidance has negatively impacted human health, potentially increasing mortality rates. For instance, social isolation, anxiety and economic concerns have severely impacted mental health [24], leading to a surge in overdoses [25], increased rates of domestic violence [26] and a predicted rise in suicides [27]. Physical health has also suffered. A UK survey reported that 50% of people gained weight during lockdown, and 30% postponed (either voluntarily or involuntarily [28]) advice or treatment for non-COVID medical issues [29], including serious conditions typically treated in emergency departments [30]. It will take more time to assess whether and how COVID-19 alters birth rates. Despite initial suggestions that lockdowns might produce a baby-boom (coronials) akin to upticks following power outages and low-severity storm warnings [31,32], a birth rate reduction seems more likely due to the economic downturn and avoidance of (or outright bans on; [33]) sex with non-household members.

## 3. Indirect effects

Predator avoidance often triggers indirect effects on species with which prey interact [34], thereby altering ecosystem structure and function [35]. An oft-cited example is that wolves shape the iconic landscape of Yellowstone via their non-consumptive effects on elk [36]. Although a few examples demonstrate that costly parasites (e.g. parasitoids) can also trigger indirect effects [37–40], such effects have not yet been described for pathogens. Furthermore, it has been suggested, but not yet demonstrated, that parasite avoidance might shape ecosystem structure and function [7,41].

The COVID-19 pandemic provides the first evidence that pathogen avoidance can trigger indirect effects on species with which hosts interact, as well as broader environmental impacts (figure 2). What is more, because humans are abundant and exert massive impacts on the planet, the magnitude of these indirect effects has been staggering. During lockdowns, a popular meme celebrated (and mocked) the idea that ‘nature is healing’ in the absence of humans [42], but a more balanced perspective indicates that the indirect effects of the virus range from positive to negative.

### (a) Positive

Human avoidance of COVID-19 may increase survival for some wildlife. For example, over short timescales, travel reductions reduced wildlife–vehicle conflict (i.e. roadkill) by 21–56% from early March to mid-April, decreasing mountain lion mortality in California [43] and doubling the ratio of live to dead amphibians on roads in Maine [44].

Altered human behaviour also changed our interactions with other infectious agents. For example, in Hong Kong and Korea, the 2019–2020 influenza epidemic period was shorter and the epidemic peak lower than in previous seasons [45,46], a phenomenon that has been echoed in the Southern Hemisphere [47]. However, COVID-19 avoidance might increase the incidence of other pathogens; Legionnaires disease is surging [48], routine vaccine administration has declined [49], and attention and resources have shifted away from other infectious diseases [50].

Reduced activity following lockdowns also generated widespread, albeit short term, reductions in air pollution and greenhouse gas emissions. One estimate suggests that by April 2020, daily global CO_2_ emissions were 17% lower than mean 2019 levels [18], and reductions in carbon monoxide, sulfur dioxide, nitrogen oxides, volatile organic compounds and particulate matter emissions were also reported [51–53]. However, these environmental benefits are temporary [54]; as normal activities resume, emissions have rebounded and could further increase due to rising car sales [55], likely driven by fears of disease exposure on public transportation, and reluctance to implement regulations that might harm struggling economies.

### (b) Negative

COVID-19 avoidance has also had negative effects on other species and the environment. For instance, in an effort to prevent infection, we are producing, using and discarding more single-use containers and personal protective equipment (PPE) than ever before. For example, mask production has dramatically increased (e.g. 200 million produced per day in China [56]), and this PPE often ends up littering natural spaces [57]. Furthermore, many regulations banning plastic bag distribution have been suspended or delayed due to the pandemic [58].

Altered patterns of food purchasing and consumption disrupted supply chains, resulting in farmers destroying crops. During peak lockdown, US farmers discarded up to 3.7 million gallons of milk each day, and a chicken processor smashed 750 000 eggs per week [59]. The environmental toll associated with disrupted supply chains has not been calculated, but given that each food calorie requires roughly 10 fossil fuel calories to produce [60], it is likely to be substantial.

Negative impacts could extend far into the future, as the COVID-19 pandemic has also distracted from and delayed environmental research, policy, management and education work. For example, research laboratories have been forced to end experiments, cancel travel and shift research priorities [61]. In the policy realm, meetings such as the United Nations Climate Change Conference (COP26) and the IUCN World Conservation Congress have been postponed, delaying plans to mitigate climate change and biodiversity loss. Finally, important management programmes have been halted [62], and many environmental education programmes have paused in-person programming [63].

### (c) Neutral

Altered human behaviour has also had cascading impacts on wildlife that (at least from a human perspective) are neither positive nor negative. For example, during lockdowns in Thailand, macaques, which normally feed on food waste discarded by tourists, fought over scraps [64]. While peridomestic species struggled, lockdowns also caused wild animals to revert to behaviours and occupy habitats that they typically avoid in the presence of humans. Although no dolphins actually swam in Venice canals [42], wild Kashmiri goats fed on hedges in a deserted town in Wales [65], and white-crowned sparrows altered their songs in newly quieted urban areas [66]. As the apex predator in many systems, humans profoundly impact animal movement, behaviour and habitat use [67,68], so it is not surprising that other species respond to our absence [69]. Indeed, the COVID-19 pandemic has created unique opportunities to study how humans impact the environment, and we are already seeing a surge in research in this area [70].

### (d) Feedback loops

In a few cases, these indirect effects have been strong enough to trigger knock-on effects, some of which involve humans (i.e. feedback loops). For instance, reduced pollution likely reduced the incidence of paediatric asthma [71], and might have contributed to fewer premature births [72]. However, not all feedback loops are beneficial. The current outbreak of *Salmonella* in the USA is likely due to the increased abundance of backyard poultry [73].

### (e) Complex socio-ecological interactions

As our disease avoidance behaviour occurs at both individual and societal levels, not all observed dynamics fit into an ecological framework. For example, changes in policy and institutional support also impact wildlife in ways that are both positive (e.g. wildlife trade bans might promote species conservation) and negative (e.g. reduced ecotourism diminishes conservation funding). Critically, while ecological theory can provide insight into human disease avoidance and its consequences, it is important to recognize that our behaviour is complex, and the ways we interact with ecosystems are often unique to our species.

## 4. Conclusion

The consumptive effects of SARS-CoV-2 have been staggering: thus far, more than 49 million have been sickened and more than 1.2 million have died. The non-consumptive effects of SARS-CoV-2 result from these consumptive effects; due to the devastating consequences of infection, humans have substantially altered their behaviour to avoid becoming infected, and evidence is accumulating that these behavioural shifts are impacting human fitness. Furthermore, disease avoidance has triggered profound indirect effects on other species and the environment, providing the first evidence that pathogens can produce such effects. From an ecological perspective, could the non-consumptive effects of the virus exceed its consumptive effects? Intuition suggests that this might be the case, because the vast majority of us have altered our behaviour, while only a small minority have (thus far) become infected. Demographers could eventually quantify this by comparing birth rates before versus after the pandemic, and deaths directly attributable to COVID-19 versus those due to other causes (in excess of baseline) [74]. For now, we can conclude that the current pandemic provides the most convincing demonstration to date that parasite avoidance, like predator avoidance, can be incredibly costly to hosts, and have cascading impacts on ecosystems. However, comparing the virus's consumptive and non-consumptive effects does not imply the existence of a trade-off between them. Regardless of government policies, individuals will continue to exhibit infection avoidance as long as the virus remains widespread in their communities. No matter how many times Donald Trump repeats that the ‘cure' cannot be worse than the disease, controlling the virus remains the only way to minimize both its consumptive and non-consumptive effects.

## Data accessibility

This article has no additional data.
